# Impact of new DAA therapy on real clinical practice: a multicenter region-wide cohort study

**DOI:** 10.1186/s12879-018-3125-6

**Published:** 2018-05-16

**Authors:** Simone Lanini, Paola Scognamiglio, Alessandra Mecozzi, Lorella Lombardozzi, Vincenzo Vullo, Mario Angelico, Antonio Gasbarrini, Gloria Taliani, Adolfo Francesco Attili, Carlo Federico Perno, Adriano De Santis, Vincenzo Puro, Fabio Cerqua, Gianpiero D’Offizi, Adriano Pellicelli, Orlando Armignacco, Francesco Saverio Mennini, Massimo Siciliano, Enrico Girardi, Vincenzo Panella, Giuseppe Ippolito, members of the Lazio Region HCV treatment group, C. Sarrecchia, M. Pompili, G. D’Ettorre, C. Pasquazzi, R. Guarisco, M. Montalbano, E. Boumis, U. Visco-Comandini, M. Zaccarelli, A. Ammassari, R. Lionetti, S. Murachelli, I. MezzaRoma, M. D. Di Paolo, C. Mastroianni, S. Francioso, C. Puoti, A. Grieco, C. Furlan, L. Loiacono, L. Fondacaro, G. Cerasari, D. Accapezzato, G. Starnini, M. Merili, S. Corradini, M. Lichtner, L. Ridola, A. Caterini, E. Tamburrini, R. Villani, L. Sarracino, S. Sereno, A. Brega, A. Antinori, M. Marignani, I. Lenci, C. Fimiani, L. Sarmati, R. Apaccini, L. Spilabotti, T. Coluzzi, K. Casinelli, F. Paoletti, V. Mercurio, C. Mastropietro, L. Miele, P. Noto, A. Moretti, P. Guarascio, C. D’Ambrosio, G. Labbadia, C. Del Borgo, U. Vespasiani, F. Palmieri, S. Cicalini, S. Cerilli, A. Sampaolesi, L. Vincenzi, R. Bellagamba, R. Cecere, V. Galati, A. Abdeddaim, G. Galati, F. Iacomi, G. Iannicelli, G. Gentile, M. Bonaventura, M. Scudieri

**Affiliations:** 1 National Institute for Infectious Diseases Lazzaro Spallanzani IRCCS, Rome, Italy; 2 Servizio Regionale per la Sorveglianza delle Malattie infettive (SeRESMI), Rome, Italy; 3 Regione Lazio Direzione Regionale Salute e Politiche Sociali, Rome, Italy; 4 Dipartimento di Sanità Pubblica e Malattie Infettive Sapienza Università di Roma, Rome, Italy; 5 Unità di Epatologia e Trapianti, Fondazione Policlinico Tor Vergata, Rome, Italy; 6 Gastroenterologia, Fondazione Policlinico Gemelli, Universita’ Cattolica del Sacro Cuore, Rome, Italy; 7 Dipartimento di Medicina Clinica Sapienza, Università di Roma, Rome, Italy; 8 Department of Experimental Medicine and Surgery, University of Roma Tor Vergata, Rome, Italy; 9 Lazio Crea, Rome, Italy; 10 UOC Malattie Infettive Epatologia Dipartimento Interaziendale Trapianti National Institute for Infectious Diseases Lazzaro Spallanzani IRCCS, Rome, Italy; 11 UOC Malattie del Fegato Dipartimento Interaziendale Trapianti AO San Camillo Forlanini Roma, Rome, Italy; 12 UOC Malattie Infettive, Ospedale Belcolle, Viterbo, Italy; 13 EEHTA CEIS, Università di Roma “Tor Vergata” e Institute of Leadership and Management in Health, Kingston University, London, UK

**Keywords:** Hepatitis C virus, Chronic hepatitis C, Liver cirrhosis, Direct acting antiviral, Multicenter cohort study, Mixed effect model, Liver damage, Treatment efficacy, Clinical study, New therapy

## Abstract

**Background:**

Management of chronic hepatitis C (CHC) has significantly accelerated in the last few years. Currently, second generation direct acting antivirals (DAAs) promise clearance of infection in most of patients. Here we present the results of the first analysis carried out on data of Lazio clinical network for DAAs.

**Methods:**

The study was designed as a multicenter cohort: a) to assess the evolution of treatment during the first 24 months of the activity of the Clinical Network; b) to report overall efficacy of treatments; c) to analyze potential factors associated with lack of virological response at 12 weeks after therapy (SVR12); d) to evaluate the variation of ALT at baseline and 12 weeks after therapy in those who achieved SVR12 in comparison to those who did not. Analyses of efficacy were carried out with multilevel mixed effect logistic regression model. ALT temporal variation was assessed by mixed effect model mixed models with random intercept at patient’s level and random slope at the level of the time; i.e. either before or after therapy.

**Results:**

Between 30 December 2014 and 31 December 2016 5279 patients started a DAA treatment; of those, 5127 (in 14 clinical centers) had completed the 12-week follow-up. Overall proportion of SVR12 was 93.41% (*N* = 4780) with no heterogeneity between the 14 clinical centers. Interruption as the consequence of severe side effect was very low (only 23 patients). Unadjusted analysis indicates that proportion of SVR12 significantly changes according to patient’s baseline characteristics, however after adjusting for potential confounders only adherence to current guidelines, stage of liver diseases, gender, transplant and HIV status were independently associated with the response to therapy. Analysis of ALT temporal variation showed that ALT level normalized in most, but not, all patients who achieved SVR12.

**Conclusion:**

Our study confirmed the extraordinary efficacy of DAAs outside clinical trials. The advantage of DAAs was particularly significant for those patients who were previously considered as difficult-to-treat and did not have treatment options before DAAs era. Intervention based on network of select centers and prioritization of patients according to diseases severity was successful. Further studies are needed to establish whether clearance of HCV after DAAs therapy can arrest or even revert liver fibrosis in non-cirrhotic patients and/or improve life quality and expectancy in those who achieve SVR12 with cirrhosis.

## Background

The hepatitis C virus (HCV) has been firstly identified in 1989 as a blood-borne human pathogen [1, 2]. Acute infection with HCV is often asymptomatic, however about 80% of infected people cannot clear the virus and develop chronic infection [3, 4]. It is estimated that chronic hepatitis C (CHC) kills about 700,000 people each year at global level [5].

Clinical approach for CHC has gradually improved over the last 30 years [6]. However, since 2014 management of CHC has been re-invented by the introduction of the direct acting antivirals (DAAs) [7]. Registered clinical trials showed that the combinations of second generation DAAs can achieve HCV clearance in most of HCV infected subjects including difficult-to-treat patients (e.g. those with cirrhosis, transplant recipients, therapy experienced patients and HIV co-infected subjects) [8]. These astonishing results have been confirmed by clinical observational studies [9–12] suggesting that currently anti-HCV therapy may be see also as a public health intervention aimed to control, and potentially, eliminate HCV [1, 7, 13, 14].

For maximizing the impact of new therapy at population level, many National Health Systems promoting universal access to care [15], have implemented DAAs into clinical practices using different strategies tailored on the local epidemiology of CHC and on the availability of economic resources. Two main strategies [16] have been proposed: a) treatment as prevention, targeted to reduce incidence of new HCV infections; b) prioritization for the stage of the disease, targeted to minimize CHC associated morbidity and mortality.

According to the Italian National Policies, since January 2015 the health authority of Lazio (an administrative Region in central Italy) has implemented a strategy for the access to DAAs based the on prioritization of patients with advanced liver diseases, extrahepatic manifestation and others severe clinical manifestations.

Here we present the results of the first analysis carried out on data of Lazio clinical network for DAA.

## Methods

### Study design and aims

This is a multicenter prospective cohort study enrolling patients with CHC who receive therapy with second generation DAAs in Lazio. Here we report the analyses carried out: A) to describe access to DAAs in the Region and to assess the resilience of the network to changing policies and guidelines; B) to assess association between treatment outcome and patient’s epidemiological and clinical characteristics; C) to evaluate temporal variation of ALT level before therapy and 12 weeks after the end of treatment.

### Setting

Lazio is an Italian Region with about 5.6 million inhabitants. About 47% of Lazio inhabitants live in Rome, the only large city. All other people live in the 347 municipalities, mainly towns (median habitants 2674 IQR 1120–7997). The Italian National Health System endorsed scheme for reimbursement of DAAs therapies based on stage of liver disease. These schemes guaranteed free access to DAAs to all subjects with CHC according to 7 criteria (five additional criteria to expand access to DAAs have been recently established) [17].

Until the end of 2016 these criteria prioritized for treatment with DAAs patients with cirrhosis (CHILD A and B) and/or resected HCC, patients with advanced liver fibrosis (Metavir F3), transplant recipients, candidates to liver transplant, patients with severe extrahepatic manifestations and patients with METAVIR F2 liver fibrosis with co-morbidities (HIV/HBV co-infections, non-viral hepatitis, diabetes, BMI ≥30, hemoglobinopathies and coagulation disorders). To implement the national reimbursement scheme and to guarantee equal access to care, Lazio Regional Health Authority has centralized DAAs supplies and created a clinical network consisting of 14 selected clinical centers for evaluation and treatment of patients with CHC. In Lazio, no patient outside this clinical network could receive DAA therapy under the national reimbursement scheme.

### Participants and follow-up

Patients were eligible for analysis if they received therapy for CHC in the Lazio and:

A. received for the first time one or more of the subsequent drugs: sofosbuvir (SOF), simeprevir (SIM), daclatasvir (DAC), ledipasvir (LED), ombitasvir+paritaprevir+ritonavir± dasabuvir (2D/3D);
B. started therapy between 30 December 2014 and 31 December 2016

For each patient, we analyzed the following information at:

A. Start of therapy: code of the clinical center; date of start of therapy; criterion for access to DAA, DAA regimen; age; gender; HCV RNA; HCV genotype; body max index (BMI); stage of liver diseases, HBsAg; anti-HIV Ab; status for previous therapy; ALT; history of OLT and of HCC;
B. end of therapy: date of end of therapy; life status; whether therapy was interrupted as consequence of severe adverse reaction;
C. 12 weeks after the end of therapy: date of data collection; HCV RNA; life status; ALT.

### Treatment outcome

Treatment was successful if patient was alive and with undetectable HCV RNA level at 12 weeks after the end of therapy (sustained virological response at 12 weeks after therapy; SVR12).

Treatment failed if at 12 weeks after the end of therapy patient either: A) had detectable HCV RNA level; B) started a new anti-HCV treatment; C) has died.

### Data sources and measurement

All data analyzed come from the Lazio Regional Network for DAAs. The network has been operational since 30 December 2014 and it was formally enforced by a Regional Act on 12 February 2015. Patient’s clinical characteristics at enrollment were analyzed as reported by doctors who evaluated patients for eligibility to national reimbursements scheme. Detection of HCV RNA in blood was performed by sensitive quantitative molecular assays with lower limit of detection as declared by manufacturer (in all cases ≤15 international units/ml); HCV genotype was determined by method able to distinguish between genotype 1a and genotype 1b. ALT level was quantified with standard laboratory methods implemented in local clinical centers.

For the purpose of this study we used the 2016 EASL guidelines [8] for defining 3 groups of quality of treatments: ***group A*,** treatments considered as optimal in 2016 EASL guidelines; ***group B,*** treatments that were shorter than recommended and/or did not include all the recommended drugs/combinations and thus were considered suboptimal in 2016 EASL guidelines; ***group C,*** treatments that contained all drugs as group A, but also included additional drug (e.g. ribavirin; RBV) and/or it were longer than recommended. This rating was meant for assessing the resilience of the network in the rapidly changing scenario and not for evaluating the performance of the individual clinical centers.

### Statistics

Association between patients’ clinical characteristics and treatment outcome (SVR12) was analyzed in bivariable and multivariable mixed effect multilevel logistic (MEML) regression models with random intercept to correct for the effect of correlation of data at level of the clinical centers. Bivariable models were used to estimate proportions of SVR12, to assess potential heterogeneity across clinical centers and to estimates unadjusted association between proportion of SVR12 and patient’s clinical features. Multivariable MEML was implemented to adjust for the effect of potential confounders using all covariates with *p* < 0.100 in the bivariable analysis. Odds-ratio (OR) for failing (i.e. the complement of SVR12) was used to describe the association between the risk of treatment failure and patient’s characteristics.

Changes of ALT at baseline and 12 after the end of therapy was assessed for the four groups of patients (i.e. non-cirrhotic patients with SVR12; non-cirrhotic patients without SVR12; cirrhotic patients with SVR12 and non-cirrhotic patients without SVR12). The estimates were calculated by a balanced (i.e. all patients had value of ALT at before and 12 weeks after therapy) linear mixed effect model with random intercept at the level of patients and random slope at the level of time. We have previously validated these model in the analysis of complex clinical datasets [18, 19]. Overall the model included ALT level as the unique continuous dependent variable and 3 independent binary variables i.e.: time (either at before therapy or 12 weeks after therapy); clinical outcome (either SVR12 or failure) and presence of cirrhosis before therapy (either yes or no) allowing for full interaction between them.

All analyses and plots were implemented by STATA 13.1 statistical package.

## Results

**Fig. 1:**
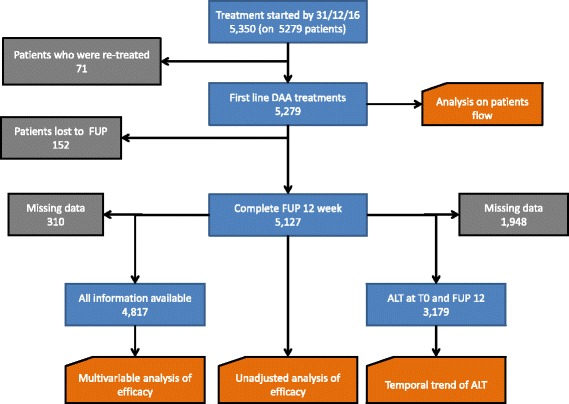
Flow charts to describe selection of population sample included in the different analyses. Blue boxes report the number of patients in each stage of selection; green boxes report excluded subjects with reason; orange boxes report the type of analysis carried out

Figure 1 shows the subsets of population included in the different analyses.

### Access to therapy with new DAA

Between 30 December 2014 and 31 December 2016 5279 patients initiated 5350 DAA treatments in Lazio. Seventy-one treatments were given as given as second line-treatment and were excluded from the analysis. Overall the analysis included all the 5279 patients at the time of their first treatment with DAAs.

**Fig. 2:**
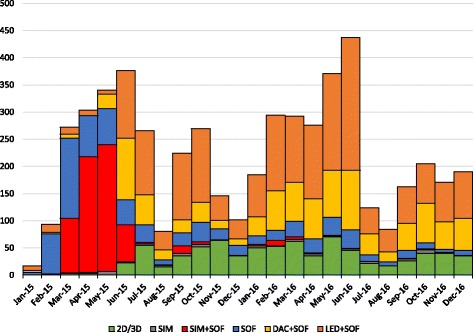
Distribution of the 5279 DAA naïve patients according to DAAs combination and month of start of therapy. SOF: sofosbuvir±ribavirin±Peg-Interferon; SIM: simeprevir+ribavirin+Peg-interferon. 2D/3D: ombitasvir+paritaprevir+ritonavir±dasabuvir±ribavirin; LED+SOF: ledipasvir+sofobuvir; DAC + SOF: daclatasvir+sofosbuvir. The frequency of each specific combination is reported in Table 1

Figure 2 shows the number of patients by the time when therapy was started and DAA combination used. Among the 5279 patients who received first line treatment with DAAs, SOF was the most used drug (87.07%; *N* = 4438) followed by LED (36.92%; *N* = 1949) DAC (20.06%; *N* = 1059), 2D/3D combinations (14.76%; *N* = 779) and SIM (13.71%; *N* = 724). RBV was used in about half of all DAA scheme (49.69%; *N* = 2623) while the use of pegylated interferon in combination either with SOF (1.48%; *N* = 78) or SIM (1.18%; *N* = 62) plus RBV was marginal and limited to the earliest period only Treatment intended duration ranged between 8 and 48 weeks, but almost all the treatments were planned for a duration of either 12 (57.81%; *N* = 3052) or 24 weeks (41.37%; *N* = 2184). Only few DAA treatments were planned for a duration of either 8 (0.45% *N* = 24), 16 (0.08% *N* = 4) or 48 weeks (0.28% *N* = 15) (Table 1).

**Table 1:** DAA treatments schemes

Drugs	Intended duration			Total
	12 weeks	24 weeks	Other	
*SIM + SOF*	362	–	–	362
*SIM + SOF + RBV*	298	2	–	300
*DAC + SOF*	403	262	–	665
*DAC + SOF + RBV*	75	319	–	394
*LED + SOF*	573	711	24^a^-	1308
*LED + SOF + RBV*	425	216	–	641
*3D*	318	–	–	318
*3D + RBV*	288	66	–	354
*2D + RBV*	58	49	–	107
*SOF + RBV*	175	500	15^b^	690
*SIM + PEG + RBV*	–	58	4^c^	62
*SOF + PEG + RBV*	77	1	–	78
Total	3052	2184	43	5279

Number of treatments according to DAAs combination and intended duration of the therapy. ^a^8 weeks (*N* = 24); ^b^48 weeks (*N* = 12) and 16 weeks (*N* = 3); ^c^16 weeks (*N* = 1) and 48 weeks (*N* = 3)

**Fig. 3:**
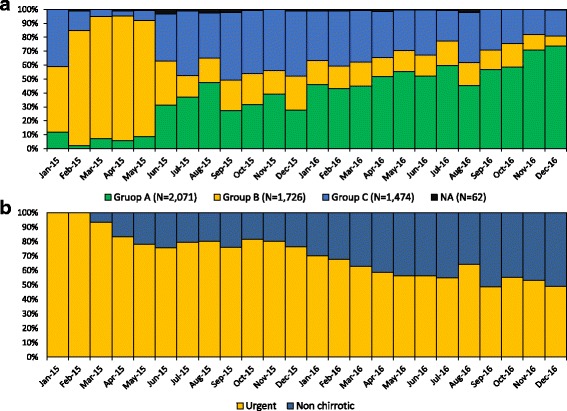
Proportion of the patients according quality of treatment and urgency of treatment. **a** Monthly proportion of the 5279 patients who received either sub-optimal, optimal or not recommend of treatment according to EASL 2016 guidelines. *Group A*, treatments currently considered as optimal in 2016 EASL guidelines; *group B,* treatments that is shorter than currently recommended and/or does not included all the recommended drugs/combinations and thus considered suboptimal in 2016 EASL guidelines; *group C,* treatments that contains all drugs as group A, but also included additional drug (e.g. ribavirin RBV) and/or it is longer than currently recommended. NA: not assessable (patients’ information to assess quality are missing). **b** Monthly proportion of the 5279 patients who had access to therapy by base line clinical features. Urgent:: patients with cirrhosis, candidate to liver transplant; recipients of organ transplant, patients with severe HCV associated extra-hepatic manifestation; Non cirrhotic: all other patients

The proportion of patients who received DAA combinations consistent with EASL 2016 guidelines was very low until May 2015 and eventually steadily increased between June 2015 and December 2016 when the 73.68% of all treatment were consistent with EASL 2016 guidelines (Fig. 3a). however, the analysis of DAA combination according to previous guidelines suggested that: A) for treatments started under EASL 2014 GL (i.e. by 21st April 2015) none could be classified as sub-optimal; B) for treatments started under EASL 2015 GL (i.e. after 22nd April 2016) 269 can be classified as sub optimal, the main reason was under use of ribavirin in cirrhotic patients in genotype 1 and 3 (*N* = 221; 82%); C) for treatments started under EASL 2016 GL (i.e. after 22nd September 2016) 89 can be classified as sub optimal, the main reason was under use of ribavirin in cirrhotic patients with genotype 3 (*N* = 30; 44%) and use of SOF + RBV in gen 2 (*N* = 28; 32%).

The proportion of patients who received DAA without cirrhosis steadily increased over time being none during the first 2 months of the study period and 51.05% by December 2016 (Fig. 3b).

### Treatment efficacy at 12 weeks after the end of therapy

The overall proportion of lost to follow-up was 2.88% (152 out of 5279 patients who started therapy). The bivariable analysis of efficacy was carried out on the 5127 patients who had completed the follow-up at 12 weeks post treatment. Overall SVR12 proportion was 93.41% (95% CI 92.48–94.34%) with no evidence of heterogeneity across clinical centers (p for heterogeneity in null model = 0.219).

In total 347 (6.59%) patients did not achieve SVR12; among them, 72 patients died by week 12 post-treatment (31 of whom during treatment) and only 23 patients interrupted the treatment for drug toxicity.

Table 2 shows descriptive analysis and the results of bivariable MEML models to assess potential association between SVR12 and individual patient’s characteristics. Proportions of SVR12 significantly change according to all analyzed patients’ baselines characteristics apart from age and previous history of treatment (range: 76.60–96.48%; see Table 2). Table 3 shows the results of multivariable MEML model to adjust for potential confounding bias. This model showed that after adjusting for other co-variates the treatment group (*p* < 0.001), stage of liver diseases (*p* < 0.001), gender (*p* < 0.001), OLT (*p* = 0.005) and HIV serostatus (*p* = 0.019) where significantly associated with SVR12. In addition, no heterogeneity for efficacy across clinical centers was observed (p for heterogeneity in multivariable MEML = 1.000).

**Table 2:** Descriptive analysis and the results of bivariable MEML models for efficacy

Patient’s clinical features		Descriptive			SVR (%)			*P*-value
		Fail	SVR12	TOT	estimate	95% CI		
Total		347	4780	5127	93.41%	92.48%	94.34%	NA
Treatment group	*group B*	182	1488	1670	89.50%	87.66%	91.34%	**< 0.001**
	*group A*	72	1891	1963	96.48%	95.58%	97.38%	
	*group C*	84	1357	1441	94.43%	93.09%	95.77%	
	*NA*	9	44	53	–	–	–	–
Cirrhosis	*no*	81	1937	2018	95.99%	95.13%	96.84%	**< 0.001**
	*CHILD A*	194	2606	2800	93.07%	92.13%	94.01%	
	*CHILD B/C*	72	237	309	76.70%	71.99%	81.41%	
	*NA*	0	0	0	–	–	–	–
Sex	*female*	85	1848	1933	95.67%	94.70%	96.65%	**< 0.001**
	*male*	262	2932	3191	91.98%	90.77%	93.18%	
	*NA*	0	0	0	–	–	–	–
Age	*≤59*	194	2474	2668	92.96%	91.73%	94.19%	0.141
	*≥60*	150	2281	2431	93.98%	92.88%	95.08%	
	*NA*	3	25	28	–	–	–	–
BMI	*BMI < 30*	288	4080	4368	93.57%	92.62%	94.53%	0.176
	*BMI ≥ 30*	59	683	742	92.25%	90.21%	94.29%	
	*NA*	0	17	17	–	–	–	–
HCV RNA a T0 (Log_10_ UI/L)	*≤ 5.99*	206	2455	2661	92.39%	91.15%	93.62%	**0.005**
	*≥6.00*	140	2306	2446	94.34%	93.33%	95.35%	
	*NA*	1	19	20	–	–	–	–
Genotype	*1a*	81	1102	1183	93.30%	91.73%	94.88%	**0.011**
	*1b*	119	1866	1985	94.09%	92.95%	95.24%	
	*2*	39	679	718	94.64%	92.94%	96.33%	
	*3*	77	728	805	90.64%	88.43%	92.84%	
	*4*	30	401	431	93.18%	90.72%	95.63%	
	*NA*	1	4	5	–	–	–	–
Previous therapy	*naive*	191	2599	2790	93.49%	92.27%	94.71%	0.758
	*experienced*	132	1876	2008	93.71%	92.41%	95.01%	
	*NA*	24	305	329	–	–	–	–
OLT	*no*	330	4662	4992	93.53%	92.63%	94.42%	**0.009**
	*yes*	17	118	135	87.75%	82.11%	93.39%	
	*NA*	0	0	0	–	–	–	–
HIV	*HIV neg.*	285	4082	4367	93.65%	92.67%	94.62%	**0.014**
	*HIV pos.*	55	527	582	90.89%	88.28%	93.50%	
	*NA*	7	171	178	–	–	–	–
HCC	*No*	324	4626	4950	93.70%	92.73%	94.67%	**< 0.001**
	*yes*	20	94	114	82.80%	75.73%	89.87%	
	*NA*	3	60	63	–	–	–	–

Descriptive and unadjusted analysis reporting the distribution of patient’s characteristics according to SVR12. 95% CI and *P*-values are calculated according multivariable mixed effect logistic model which take into account variability due to data correlation at 14 clinical centers. *BMI* body max index, *OLT* orthotopic liver transplant, *HCC* hepatocellular carcinoma, *NA* not available

**Table 3:** Multivariable MEML model for efficacy

Patients’ clinical features		Odds ratio for failure			*P*-value
		estimate	95% CI		
Treatment group	*group B*	2.62	1.91	3.58	**< 0.001**
	*group A*	Base	–	–	
	*group C*	1.23	0.85	1.77	
Cirrhosis	*no*	Base	–	–	**< 0.001**
	*CHILD A*	1.56	1.15	2.11	
	*CHILD B/C*	5.39	3.64	7.98	
Sex	*female*	Base	–	–	**< 0.001**
	*male*	1.84	1.40	2.43	
HCV RNA a T0 (Log_10_ UI/L)	*≤ 5.99*	Base	–	–	0.173
	*≥6.00*	0.84	0.66	1.08	
Genotype	*1a*	1.38	0.89	2.14	0.252
	*1b*	1.44	0.96	2.16	
	*2*	Base	–	–	
	*3*	1.61	1.05	2.48	
	*4*	1.48	0.86	2.57	
OLT	*no*	Base	–	–	**0.005**
	*yes*	2.26	1.27	4.01	
HIV	*HIV neg.*	Base	–	–	**0.019**
	*HIV pos.*	1.48	1.07	2.06	
HCC	*no*	Base	–	–	0.085
	*yes*	1.61	0.94	2.76	

Multivariable mixed effect logistic model to assess association of failing to achieve SVR12 after adjusting for potential confounders. The model has been set by including patients’ characteristics with *p*-value < 0.100 at unadjusted analysis (Table 2). 95% CI and *P*-values are calculated according considering variability due to data correlation at 14 clinical centers. *OLT* orthotopic liver transplant, *HCC* hepatocellular carcinoma

### Temporal variation of ALT level before therapy and at 12 weeks after the end of treatment

**Fig. 4:**
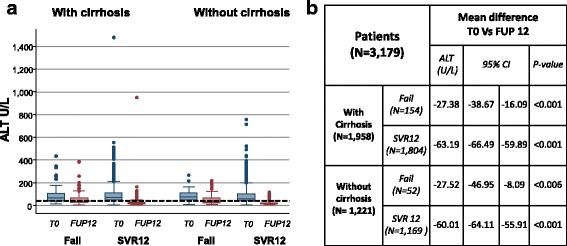
Kinetics of ALT level in 4 different groups of patients according to the stage of liver disease (i.e. with/without cirrhosis) and therapy outcome (i.e. fail or SVR12). **a** Box-plot describe the distribution of the 3179 patients according values of ALT level patients either before the start of therapy (blue boxes; T0) and eventually 12 weeks after therapy (red boxes; FUP12). Almost all patients had ALT value above upper normal limit (black dotted line; 40 U/L) before therapy. More than 75% of patients who achieve SVR12 normalized ALT at 12 weeks after the end of therapy. A normalization of ALT values was also reported in about 50% of those who did not achieve SVR at 12 weeks after the end of therapy. **b** Temporal variation of ALT levels before treatment and 12 weeks after the endo of therapy. Estimates, 95%CI and *p*-values were carried out according to a mixed effect model which take in account correlation of data at the level of each individual patients. A significant reduction of ALT is reported for all patients. However average reduction of ALT levels is about 2 time higher in those who achieved SVR12 than in those who did not

Figure 4 shows the temporal variation of ALT level before therapy and 12 weeks after the end of treatment in the four different classes of patients; i.e.: patients with cirrhosis who did not achieve SVR12; patients with cirrhosis who achieved SVR12; patients without cirrhosis who did not achieve SVR12; patients without cirrhosis who achieved SVR12. This analysis was carried out on a convenient sample of 3179 subjects who had ALT determination both before start of therapy and at the end of the follow-up.

Figure 4a shows the distribution of ALT levels for the 3179 patients included in the analysis (overall 6358 ALT determinations). ALT levels were above the upper normal limit (40 U/L) in more than 75% of patients before treatment (blue boxes in Fig. 4a). In contrast, at 12 weeks after therapy, ALT levels normalized in more than 75% of patients with SVR12 and in about 50% of those who failed, regardless the stage of liver diseases, (red boxes in Fig. 4a).

Average reduction of ALT levels before therapy and 12 weeks after therapy, 95% CI and *p*-value according to stage of liver diseases (i.e. either with or without cirrhosis) and outcome of therapy (i.e.: either SVR12 or fail) are reported in Fig. 4b. This analysis provides strong evidence that ALT significantly decreased in all patients. However, the degree of reduction was more than 2 times higher in those who achieved SVR12 than in those who did not.

## Discussion

To our knowledge, this is one of the largest prospective observational study carried out using real clinical data of patients treated with second generation DAAs. Real world studies like this are pivotal to assess the actual impact of new therapies on real clinical practice and to confirm efficacy and safety of new drugs outside clinical trials.

We showed that Lazio clinical network was capable of timely dealing with the changing guidelines and the ongoing process of approval drug. Newly DAAs were included in treatment with no delay, besides, the adherence to current standard of care steadily increased over time following to the publication of new guidelines. In addition, the number of patients who started therapy with cirrhosis steadily decreased over time, suggesting that the patients with advanced liver disease and replicating HCV infection have been steadily decreasing. For these reasons, since April 2017 the Italian policies for reimbursement of DAAs have been extended to include several sub-groups of patients in addition to those with urgent need of therapy [17].

We found that overall efficacy of DAAs therapies was well above 90% (i.e.: 93.41% with a 95% CI between 92.48 and 94.34%) which is within the efficacy range reported in clinical trials [20]. A large real world clinical study carried out in USA enrolling 4365 patients with HCV genotype 1 infection reported slightly lower efficacy rate with SVR12 between 91.3 and 92.0% [21]. This marginal difference could be due to differences in baseline characteristics; the USA study includes only genotype 1 and about 36.5% of African-American patients, in whom SVR12 rates were significantly lower than in European ones (89.8% vs. 92.8%). In addition, better proportion of SVR12 in our study compared to the USA cohort may be due to different patient management, as acknowledged by Calleja et al. [22] Indeed, multicenter prospective cohort studies carried out in other European settings, such as Spain [23] and Germany [24, 25], reported proportions of SVR similar to those observed in our study. In our clinical network, patients were managed by experienced clinicians in selected clinical centers rather than in community-based practice, which may have resulted in better adherence to treatment and consequently higher SVR12 rate [26]. The circumstance that our patients received homogeneously high quality of care throughout the Region was confirmed by no heterogeneity of efficacy across clinical centers and the very low level of lost to follow-up.(2.88%) Indeed, lost to follow-up proportion was more than 2 times lower than that reported in USA (6.80%) and comparable to those observed in similar clinical setting in Spain (2.71%) [23] and Germany (4.89%) [24].

After adjusting for other confounders, we found that quality of treatment, stage of liver diseases, gender, OLT and HIV serostatus were independent predictors of SVR12.

Our study includes a considerable number of patients who received a quality of treatment currently considered as either sub-optimal (group B) or not recommended (group C) [8]. This mirrors the dynamic real-world scenario analyzed, where standard of care changed according to the availability of additional DAAs and new clinical recommendations. In Italy SOF became reimbursable from December 2014, SIM from February 2015, DAC from April 2015 and SOF + LDV, 2D and 3D from May 2015. When the clinical network was established, effective EASL guidelines were those available since May 2014 [27] which were updated on April 2015 [28] and eventually on September 2016 [8]. As in other prospective studies carried out in Italy [10], our study provided strong evidence that adherence to the most current standards of care was associated with higher proportions of SVR12 than receiving DAAs combinations which were eventually considered sub-optimal. In addition, we did not find significant association between failure and not recommended, though not suboptimal, treatments, as those included in group C.

Cirrhosis remains a significant challenge in the DAAs era. Although, the observed efficacy of DAA therapies was much higher than that expected for interferon plus ribavirin therapies [6], unadjusted analysis showed that SVR12 dropped from 95.99% in patients without cirrhosis to 76.70% in those with decompensated cirrhosis. Multivariable analysis confirmed this observation and provided evidence that, in comparison with patients without cirrhosis, the risk of failing SVR12 was 1.56 and 5.39 times higher in patients with either compensate or decompensate cirrhosis, respectively. Unadjusted proportions of SVR12 in patients with cirrhosis were slightly lower in our population than those estimated in other studies where patients with cirrhosis received SOF in combination with either DAC [29–31] or LED [30–32]. The difference may be due to the unavailability of such regimens in the earliest period of the study, when SOF in combination with RBV was the only available combination.

We found that females exhibited higher proportion of SVR12 than males. The role of gender as predictor of response to anti-HCV therapies has been debated since long before DAAs introduction. Population studies provided evidence that females were more likely than males to naturally clear HCV after infection [33]. Clinical studies suggested that young females had better response to interferon therapy than males [34–36] but menopause may abolish this advantage [37]. Recent clinical experiences with DAAs seem to confirm this observation [24, 38], however whether a causal (biological) link stands behind the association between gender and response to anti HCV therapies is unclear, yet. The explanation of the potential biological pathways of the association between SVR12 and gender is beyond the scope of this study. Nevertheless, it is worth of notice that our estimate (OR 1.84 95% CI 1.40–2.43) is widely consistent with those reported in similar large prospective studies carried out in Europe [23, 24, 39, 40].

We found that HIV serostatus was an independent predictor of SVR12. The potential effect of HIV serostatus on efficacy of new DAAs therapies is currently debated. Bischoff et al. [39] found that HIV positive and HIV negative subjects had similar proportion of SVR12 (90.3 and 91.2%, respectively). However, these results may be the consequence of confounding as prevalence of cirrhosis was significantly higher among HIV negative than among HIV positive subjects (29.5% vs 17.2%, respectively; *p* < 0.001) and authors did not adjust statistics for the level of liver diseases in the multivariable analysis. In contrast, Neukam et al. [41], reported that, after adjustment for potential confounders, including cirrhosis, HIV positivity was associated with lower proportion of SVR12. Moreover, recent studies specifically focused on HIV positive population reported proportions of SVR consistent to those observed in our study and emphasize that level of CD4 is directly associated with SVR12 [40]. Consistently with these latter experiences our study suggests that, even if DAAs are very effective in HIV positive subjects (with SVR12 portion higher than 90%), patient with HIV may still have marginally lower proportion of SVR12 than those without HIV infection.

We found a proportion of SVR among OLT recipients of 87.75% (95 82.11–93.39%) which is significantly lower than SVR in subjects without OLT. This is one of the first large prospective study which attempted to assess association between OLT and SVR. Our observation is consistent with punctual estimates of SVR proportion reported in prospective studies enrolling OLT patients only [42, 43].

Finally, our study provided strong evidence that DAAs therapy was associated with a significant reduction of liver cytolysis. The role of DAAs in the reduction of liver damage has been already suggested by a previous study [44]. However, we found that ALT levels normalized in most, but not all, patients who achieved SVR12, implying that hepatic inflammation may continue in a sub set of patients despite HCV clearance [45]. This finding also suggest that patients who achieved SVR12 would need long-term clinical follow-up to monitor the improvement of liver diseases and further underlines that HCV infection may be associated with comorbidities that on one hand contribute to the progression of the disease and, on the other hand, need to be managed once HCV has been eradicated.

Limitation of our study are related to: A) observational design, thus confounding due to unmeasured exposures cannot be ruled out; B) even though the information were collected through a compulsory system enforced by Regional laws, we still had a moderate proportion of missing data; C) analysis to assess the effect of SVR on hepatic cytolysis was carried out on convenient sub set of patient for whom ALT determinations were available; D) we do not systematically collect data on resistance before therapy, this limitation may be marginal for the purposes of our analyses as current guidelines does not recommend resistance testing prior to treatment with DAAs and acknowledge that treatment regimens can be optimized without this information [8].

## Conclusion

In conclusion, our study confirmed the extraordinary efficacy of DAA therapies outside clinical trials and provided evidence that adherence to most current guidelines can further improve the response rate in all patients. The advantage of using DAAs was particularly significant for those patients who were previously considered as difficult-to-treat and had no-treatment option before DAAs era. As in other European experiences, our model of intervention based on the creation of a network of select clinical centers and prioritization of patients according to diseases severity was successful and guaranteed timely implementation of new drug combinations, high level of SVR12 and the significant reduction of patients with advance stage CHC in urgent need of therapy. Our study analyzed as the only outcome clearance of HCV infection (i.e. SVR12) thus it provides no information about the actual impact of DAAs therapy on patient’s life quality and expectancy after SVR. Further studies are needed to establish whether clearance of HCV after DAAs therapy can arrest, or even revert, liver fibrosis in non-cirrhotic patients and/or improve life expectancy in those who achieved SVR12 with advanced stage live disease [46]. In our opinion, long term clinical follow-up is needed also in patients who achieved SVR12.

## Abbreviations

2D: Ombitasvir+paritaprevir+ritonavir
3D: Ombitasvir+paritaprevir+ritonavir+dasabuvir
ALT: Alanine aminostransferase
BMI: Body max index
CHC: Chronic hepatitis C
DAA: Direct acting antivirals
DAC: Daclatasvir
EASL: European Association for the study of the liver
HCC: Hepatocellular carcinoma
HCV: Hepatitis C virus
HIV: Human immunodeficiency virus
LED: Ledipasvir
MEML: Mixed effect multilevel logistic
OLT: Orthotopic liver transplant
RBV: Ribavirin
RNA: Ribonucleic acid
SIM: Simeprevir
SOF: Sofosbuvir
SVR12: Sustained virological response at 12 weeks after therapy
